# Long-term analysis of humoral responses and spike-specific T cell memory to Omicron variants after different COVID-19 vaccine regimens

**DOI:** 10.3389/fimmu.2024.1340645

**Published:** 2024-03-12

**Authors:** Chia-Lo Ho, Li-Chen Yen, Hong-Wei Huang, Chun-Chi Lu, Yi-Jen Hung, Ching-Len Liao, Chin-Mao Hung, Kuo-Chou Chiu

**Affiliations:** ^1^ Institute of Life Sciences, National Defense Medical Center, Taipei, Taiwan; ^2^ Department of Microbiology and Immunology, National Defense Medical Center, Taipei, Taiwan; ^3^ Division of Rheumatology/Immunology and Allergy, Department of Internal Medicine, Tri-Service General Hospital, National Defense Medical Center, Taipei, Taiwan; ^4^ Division of Endocrinology and Metabolism, Department of Internal Medicine, Tri-Service General Hospital, National Defense Medical Center, Taipei, Taiwan; ^5^ National Institute of Infectious Diseases and Vaccinology, National Health Research Institutes, Miaoli, Taiwan; ^6^ Institute of Preventive Medicine, National Defense Medical Center, Taipei, Taiwan; ^7^ Division of General Dentistry, Taichung Armed Forces General Hospital, Taichung, Taiwan; ^8^ School of Dentistry, China Medical University, Taichung, Taiwan; ^9^ School of Dentistry, National Defense Medical Center, Taipei, Taiwan

**Keywords:** SARS-CoV-2, Omicron variant, vaccination, neutralizing antibody, cellular immune response

## Abstract

**Background:**

The emergence of SARS-CoV-2 variants has raised concerns about the sustainability of vaccine-induced immunity. Little is known about the long-term humoral responses and spike-specific T cell memory to Omicron variants, with specific attention to BA.4/5, BQ.1.1, and XBB.1.

**Methods:**

We assessed immune responses in 50 uninfected individuals who received varying three-dose vaccination combinations (2X AstraZeneca + 1X Moderna, 1X AstraZeneca + 2X Moderna, and 3X Moderna) against wild-type (WT) and Omicron variants at eight months post-vaccination. The serum antibody titers were analyzed by enzyme-linked immunosorbent assays (ELISA), and neutralizing activities were examined by pseudovirus and infectious SARS-CoV-2 neutralization assays. T cell reactivities and their memory phenotypes were determined by flow cytometry.

**Results:**

We found that RBD-specific antibody titers, neutralizing activities, and CD4+ T cell reactivities were reduced against Omicron variants compared to WT. In contrast, CD8+ T cell responses, central memory, effector memory, and CD45RA+ effector memory T cells remained unaffected upon stimulation with the Omicron peptide pool. Notably, CD4+ effector memory T cells even exhibited a higher proportion of reactivity against Omicron variants. Furthermore, participants who received three doses of the Moderna showed a more robust response regarding neutralization and CD8+ T cell reactions than other three-dose vaccination groups.

**Conclusion:**

Reduction of humoral and CD4+ T cell responses against Omicron variants in vaccinees suggested that vaccine effectiveness after eight months may not have sufficient protection against the new emerging variants, which provides valuable information for future vaccination strategies such as receiving BA.4/5 or XBB.1-based bivalent vaccines.

## 1 Introduction

In order to control the spread of the COVID-19 pandemic, vaccination has emerged as an essential strategy to mitigate the transmission and severity of SARS-CoV-2 infections. Vaccines have demonstrated remarkable efficacy in preventing infection, reducing the disease’s mortality, and minimizing disease complications (1, 2). Several COVID-19 vaccines have been approved for administration in Taiwan, including the mRNA vaccine Spikevax (mRNA-1273; Moderna [here referred to as Mod]) and the viral vector-based vaccine Vaxzevria (ChAdOx1-nCoV-19; AstraZeneca [here referred to as AZ]), both of which were authorized for homologous or heterologous prime-boost regimens elsewhere.

However, the emergence of variants of concern (VOCs), especially the Omicron variants, has substantially threatened the vaccine efficacy. Mutations in the viral genome have led to the emergence of variant strains with altered spike protein structures, potentially reducing the efficacy of neutralizing antibodies generated by previous vaccines (3–5). Numerous studies have demonstrated that the Omicron variant exhibits an unprecedented escape from neutralizing antibodies, affecting both convalescent and vaccinated populations (6–10). In order to compete with the decrease in antibody levels over time and the ongoing evolution of SARS-CoV-2 variants, individuals in Taiwan were encouraged to receive a third dose of the mRNA vaccine. After administering the third dose, serum anti-spike antibody levels and neutralization titers against SARS-CoV-2 increased, although to a lesser degree against the Omicron variant (11–14). This necessitates a more comprehensive evaluation of the long-term immune responses induced by triple vaccinations, encompassing both humoral and cellular immunities, especially for the circulating Omicron sublineages BA.4/5, BQ.1.1, and XBB.1.

While humoral immunity is effective in preventing infection, it may not be sufficient alone to counter SARS-CoV-2 variants, which have the potential to evade neutralizing antibodies (15). Cellular immunity, mediated by T cells, is an essential arm of the immune system that can provide broad and long-lasting protection against viral infections (16). Previous studies have shown that robust CD4+ and CD8+ T cell responses and their memory subsets were induced following SARS-CoV-2 vaccination (14, 17–20). However, it is still not fully understood whether the long-term levels of spike-specific T-cell responses and the induction of memory phenotypes were affected against emerging Omicron variants in populations receiving three vaccine doses.

In this study, we evaluated the levels of RBD-specific antibody titers, neutralizing activities, spike-specific T cell reactivities, and their memory subsets for up to 8 months against SARS-CoV-2 WT and Omicron variants in three-dose vaccination groups, including AAM (2X AZ + 1X Mod), AMM (1X AZ + 2X Mod), and MMM (3X Mod). Both adenovirus vector–based (AZ) and mRNA-based (Mod) vaccines use the SARS-CoV-2 S protein from the ancestral strain, eliciting strong immune responses and providing protection against severe COVID-19 (21). Including AZ and Mod vaccines in different combinations allows for exploring hybrid immunity strategies, capitalizing on the strengths of different vaccine platforms. As SARS-CoV-2 variants continue to emerge, knowing the complexities of long-term vaccine-induced immunity in vaccinees becomes crucial for revising vaccination strategies and compositions and as we strive to curb the ongoing pandemic.

## 2 Materials and methods

### 2.1 Cell lines and viruses

Baby hamster kidney (BHK)-21, a BHK cell line (ATCC CCL-10), cells were cultured in Roswell Park Memorial Institute 1640 medium containing 5% fetal bovine serum (FBS) (Gibco). The BHK-21 cells were stably transduced with the lentiviral vector harboring angiotensin-converting enzyme (ACE2) and selected with puromycin (Sigma) at 10 μg/mL to obtain BHK-ACE2 cells, as described previously (22). Human embryonic kidney 293 (HEK293), an HEK cell line (ATCC CRL-1573), cells were grown in Dulbecco’s Modified Eagle Medium (DMEM) supplemented with 10% FBS. SARS-CoV-2 The SARS-CoV-2 WT strain (hCoV-19/Taiwan/4/2020) and B.1.1.529.1 (hCoV-19/Taiwan/16804/2021, BA.1 variant), and B.1.1.529.5 (hCoV-19/Taiwan/689423/2022, BA.5 variant) were provided by the Taiwan Centers for Disease Control, Ministry of Health and Welfare, and propagated using Vero E6 cells supplemented with 2% FBS. Passage-3 virus was used for all the studies described here. Viral stocks were contamination-free, and viral titers were determined by plaque assay, followed by storage of aliquots at −80°C until further use in experiments.

### 2.2 Participants

Fifty adult participants (aged 27–63 years) with good physical health (mild-to-moderate well-controlled comorbidities were permitted) were enrolled between March 2022 and December 2022 in the Tri-Service General Hospital in Taiwan. According to the primed COVID-19 vaccines they had received, the participants were subdivided into three groups: AAM (n = 18), AMM (n = 16), and MMM (n=16). Participants in AAM had received two standard prime doses of AZ (0.5 mL/dose containing 5 × 10^10^ viral particles via intramuscular injection) followed by a single dose of Mod (100 μg administered at 0.5 mL via intramuscular injection). The median durations between the two doses of AZ and the single booster of Mod were 35 and 89 days, respectively. Participants in AMM had received one standard prime dose of AZ (0.5 mL/dose containing 5 × 10^10^ viral particles via intramuscular injection) followed by two doses of Mod (100 μg administered at 0.5 mL via intramuscular injection). The median durations between the one dose of AZ and two doses of Mod were 80 and 92 days, respectively. Participants in MMM had received one standard prime dose of Mod (100 μg administered at 0.5 mL via intramuscular injection) followed by two doses of Mod (100 μg administered at 0.5 mL via intramuscular injection). The median durations between the one dose of Mod and two doses of Mod were 80 and 92 days, respectively. Blood samples were collected 180-190 days after receiving the three vaccine doses.

### 2.3 Enzyme-linked immunosorbent assay

Serum SARS-CoV-2 anti-receptor-binding domain (RBD) and anti-nucleocapsid immunoglobulin G (IgG) antibodies were assessed by enzyme-linked immunosorbent assay (ELISA) as previously described (23). In brief, ninety-six-well plates were coated with SARS-CoV-2 RBD and Nucleocapsid protein (5 μg/mL), incubated at 4°C overnight: (1) wild-type (WT): SARS-CoV-2 (COVID-19) Spike RBD protein, His tag (active) (GTX136090-pro, GeneTex), (2) BA.1: SARS-CoV-2 (COVID-19) Spike RBD Protein, Omicron/BA.1 variant, His tag (GTX136716-pro, GeneTex), (3) BA.2: SARS-CoV-2 (COVID-19) Spike RBD Protein, Omicron/BA.2 variant, His tag (GTX136905-pro, GeneTex), (4) BA.4/5: SARS-CoV-2 (COVID-19) Spike RBD Protein, Omicron/BA.4/5 variant, His tag (GTX137098-pro, GeneTex), (5) BQ.1: SARS-CoV-2 (COVID-19) Spike RBD Protein, Omicron/BQ.1 variant, His tag (GTX137879-pro, GeneTex), (6) XBB.1: SARS-CoV-2 (COVID-19) Spike RBD Protein, Omicron/XBB.1 variant, His tag (GTX138115-pro, GeneTex), and (7) Nucleocapsid: SARS-CoV-2 (COVID-19) Nucleocapsid Protein, His tag (GTX135592-pro, GeneTex). After incubation, the plates were washed once with wash buffer and blocked with 10% bovine serum albumin (BSA) (Cyrusbioscience) per well for 2 h at room temperature. Heat-inactivated serum was serially diluted fourfold with 1% BSA at 1:50 and added to the wells, and the plates were incubated for 2 h at room temperature. After washing, anti-human IgG-horseradish peroxidase (1:100,000) (GTX26759, GeneTex) was added to the plates and incubated for 1 h at room temperature. After washing, 3,3’,5,5’-tetramethylbenzidine (TMB) substrate (Invitrogen) was added to each well and incubated for 5 min, and the reaction was stopped with 1 M sulphuric acid. Optical density (OD) was measured at 450 nm using an ELISA reader (BioTek). Endpoint titers were established using a nonlinear sigmoidal four-parameter logistic (4PL) model curve fit to calculate the reciprocal serum dilution that reached the OD450 values of the pre-pandemic sera + 3 SD.

### 2.4 Spike plasmid cloning and SARS-CoV-2 pseudovirus production

A pseudovirus carrying the variant of concern (VOC) spike protein of SARS-CoV-2 was constructed, as previously described. In brief, 60-μL Lipofectamine 3000^®^ transfection reagents (ThermoFisher) were mixed with 500 μL serum-free DMEM, sat at room temperature for 5 min, and subsequently mixed with the following three DNA plasmids that were diluted in 500 μL serum-free DMEM for another 20 min: pLAS3w-FLuc-Ppuro (9.5 μg), pCMV-Δ8.91 (Gag-Pol provider, 6.5 μg), and the indicated spike plasmids (4.5 μg): pcDNA3.1_spike_del19 (Addgene #155297), SARS-CoV-2 Omicron Strain S gene Human codon_pcDNA3.1(+) (B.1.1.529/BA.1) (GenScript # MC_0101274), pcDNA3.3 SARS2 Omicron BA.2 (Addgene #183700), pCAGGS SARS-CoV-2 BA.4/5 Spike (Addgene #186031), pCAGGS SARS-CoV-2 BQ.1.1 Spike (Addgene #193710), and pCAGGS SARS-CoV-2 XBB Spike (Addgene #195287). This DNA–lipofectamine mixture was co-transfected to HEK-293T cells (4 × 106 cells per 10-cm dish) and incubated at 37°C in a 5% carbon dioxide incubator. After overnight incubation for 16 h, the transfected cells were replenished with fresh medium for subculture. At 48 h post-transfection, the pseudovirus containing culture medium was collected by centrifugation at 1,000 × g for 10 min to removes unwanted cells or large debris, followed by passing the clarified medium through a 0.45-μm filter (Millipore Corporation. Billerica, MA, USA). Virus can be stored at 4°C for immediate use or frozen at –80°C. Pseudovirus titers were determined using a p24 ELISA kit (Takara Bio).

### 2.5 Neutralization assay with pseudotyped SARS-CoV-2 (pVNT50)

BHK-21/ACE2 cells were seeded at a density of 4 × 10^4^ cells/well in 24-well plates 16 h before the experiment. For neutralization assay, 40 μL of heat-inactivated sera was started with a 1:16 dilution in complete medium containing 2% FBS, followed by two-fold serial dilutions in duplicate samples, and then incubation with 40 μL of pseudovirus (1 ng p24) for 1 h at 37°C. On the day of infection, the cells were washed twice with PBS, and 100 μL of serum–virus mixture was added to the cells and incubated for 48 h. The cells were quenched by adding 100 μL of Bright-Glo luciferase substrate (Promega) directly to each well, and the luciferase activity was measured using a Synergy H4 luminometer (BioTek). Background values, monitored from uninfected cells, were consistently below 400 relative luminescence units, and pre-pandemic sera were used as the negative control for the neutralization assay. Sera diluted at 1:16 provided results in the range of the background relative to light unit levels. A pVNT50 > 1:16 serum dilution was regarded as positive.

### 2.6 Neutralization assay with infectious SARS-CoV-2 (PRNT50)

Serum samples were heated at 56°C for 30 minutes to inactivate complement; two-fold serial dilutions, starting at a concentration of 1:5, were mixed with an equal volume of viral solution containing 100 PFU of SARS-CoV-2. The serum-virus mixture was incubated for 1 hour at 37°C in a humidified atmosphere with 5% CO2. After incubation, the mixture at each dilution was added to Vero E6 cells and incubated at 37°C for 1 hour. Cells were subsequently cultured with DMEM containing 2% FBS and 1.4% methylcellulose for 72 hours. After culturing, plaques were stained and counted. Neutralizing antibody titers were defined as the reciprocal of the maximum dilution of serum that reduced the virus titer by 50% compared to the negative control sera, and PRNT50 below 1:5 serum dilution was considered negative.

### 2.7 Isolation of peripheral blood mononuclear cells

Peripheral blood mononuclear cells (PBMCs) derived from the participants were isolated from anticoagulant-treated whole blood using Ficoll-Paque™ PLUS density gradient medium (Cytiva #17144003) as previously described (23). For isolate PBMCs, blood diluted with PBS was gently layered over an equal volume of Ficoll in a Falcon tube and centrifuged for 30 min at 400 × g without braking. The PBMCs were gently removed using a Pasteur pipette and added to a warm medium or PBS to remove any remaining platelets. The pelleted cells were counted, and the percentage viability was estimated using trypan blue staining. The isolated PBMCs were stored in liquid nitrogen until use in assays.

### 2.8 Activation-induced marker assay

Cryopreserved Peripheral blood mononuclear cells (PBMCs) were thawed and washed with RPMI 1640 medium containing human AB serum (Sigma-Aldrich), L-glutamine (Gibco), and HEPES buffer (Gibco). The cells were then plated in U-bottom 96-well plates at 1 × 10^6^ cells per well and incubated overnight. PBMCs were stimulated with peptide pools of SARS-CoV-2 spike WT (PepTivator^®^ SARS-CoV-2 Prot_S, Miltenyi Biotec), BA.1 variant (PepTivator^®^ SARS-CoV-2 Prot_S B.1.1.529/BA.1 Mutation Pool, Miltenyi Biotec), or BA.5 variant (PepTivator^®^ SARS-CoV-2 Prot_S B.1.1.529/BA.5 Mutation Pool, Miltenyi Biotec), along with co-stimulation antibodies. Positive control cells were stimulated with phytohemagglutinin (ThermoFisher), and negative control cells were treated with DMSO in PBS.

### 2.9 Flow cytometry analysis

After 24 hours of stimulation, PBMCs were washed and stained with Zombie Red Fixable Viability Dye (BioLegend), followed by staining with antibodies against specific markers. The markers included anti-hCD3 BV510, anti-hCD4 BV605, anti-hCD8 FITC, anti-hCD134 (OX40) BV421, anti-hCD137 APC, anti-hCD69 PE-Cy7, anti-hCD197 (CCR7) PerCP-Cy5.5, and anti-hCD45RA APC-Cy7 (BioLegend). Flow cytometry analysis was conducted using the Attune™ NxT Flow Cytometer, and the gating strategy can be found in **Supplementary Figure 3**.

### 2.10 Enzyme-linked immunosorbent spot assay

The amount of antigen-specific interferon (IFN)-γ- or interleukin (IL)-2-secreting T cells was evaluated by enzyme-linked immunosorbent spot (ELISpot) assays as previously described (22). The mean SFC value of duplicate peptide pool-stimulated PBMCs was calculated and normalized by subtracting the mean of the negative control replicates (control medium), and the cut-off value for background T-cell responses was defined as the mean SFC value of seronegative PBMCs derived from healthy unvaccinated donors + 3 SD (8.5 SFC/106 PBMCs). The results are expressed as SFC per million PBMCs.

### 2.11 Ethics

This study was approved by the Institutional Review Board of Tri-Service General Hospital (TSGHIRB No. B202105097). Informed consent was obtained from all the enrolled participants. Work with infectious SARS-CoV-2 has been approved by the Institutional Biosafety Committee (IBC) and was performed in the high biocontainment Biosafety Level 3 (BSL-3) facilities of the Institute of Preventive Medicine (IPM), National Defense Medical Center (NDMC), which are approved for such use by the Taiwan Centers for Disease Control, under license D1-109-0030#1123 and D1-111-0017#2028 to institutional guidelines.

### 2.12 Statistical analyses

Statistical analyses were performed using GraphPad Prism version 5. Anti-RBD IgG titers and pVNT_50_ values are presented as medians and IQRs. A nonlinear sigmoidal 4PL model was used to determine the endpoint titers of anti-RBD IgG and pVNT_50_ in each serum sample. The statistical significance of the endpoint titers and pVNT_50_ was measured among experiments using a one-way analysis of variance (ANOVA) with Tukey’s multiple comparison test. One-way ANOVA performed the statistical significance of the T cell AIM assay with a Tukey’s *post-hoc* test for multiple pairwise comparisons.

## 3 Results

### 3.1 Participants characteristics

Blood samples were collected from 50 vaccinated individuals between March 2022 and December 2022. In group AAM, 10 (55.6%) were male and 8 (44.4%) were female, with a median age of 43.5 years. In group AMM, 8 (50%) were male and 8 (50%) were female, with a median age of 44.5 years. In group MMM, 9 (56.3%) were male and 7 (43.7%) were female, with a median age of 43.5. Only 38 participants above were involved in the T cell AIM assay. All 50 participants were monitored to be uninfected by using anti-nucleocapsid ELISA. Detailed information can be found in **Table 1**.

**Table 1:** Demographic and clinical characteristics of participant enrolled in this study.

Gender	Age	Underlying disease	Vaccination schedule^a^	Intervals between 2nd/3rd dose (days)	Collected after third dose (days)	Nucleocapsid ELISA
M	54	Hypertension	AAM	74/92	241	Negative
F	27			78/95	243	Negative
M	38			72/88	238	Negative
F	56	Hyperlipidemia		75/95	246	Negative
M	46			81/98	244	Negative
F	53			71/94	250	Negative
M	58	Hypertension		77/89	242	Negative
F	45			87/86	240	Negative
M	41			89/91	245	Negative
M	34			83/92	235	Negative
F	32			88/90	238	Negative
F	35			79/87	248	Negative
M	51	Hyperlipidemia		85/97	246	Negative
M	42			84/94	249	Negative
F	37			71/87	237	Negative
M	46			78/90	236	Negative
F	31			84/95	244	Negative
M	56	Hypertension		77/91	246	Negative
M	54		AMM	76/93	241	Negative
M	45			73/90	244	Negative
F	31			88/91	242	Negative
F	55	Hypertension		85/89	236	Negative
M	44			78/91	242	Negative
F	59	Hypertension		83/90	240	Negative
M	34			79/86	237	Negative
F	63	Hyperlipidemia		81/97	246	Negative
F	57			82/94	238	Negative
M	38			80/91	250	Negative
F	59	Hypertension		79/86	245	Negative
F	36			82/97	239	Negative
M	49			80/87	248	Negative
F	39			71/86	245	Negative
M	41			83/90	236	Negative
M	36			82/96	242	Negative
M	44		MMM	34/88	249	Negative
F	54			31/89	243	Negative
M	34			35/91	242	Negative
F	57	Hyperlipidemia		29/94	138	Negative
M	35			33/90	248	Negative
F	37			34/93	247	Negative
F	43			39/97	237	Negative
M	51			31/91	236	Negative
F	47			32/86	240	Negative
M	58	Hyperlipidemia		37/96	241	Negative
M	34			36/93	249	Negative
F	31			36/89	238	Negative
M	59	Hypertension		40/94	244	Negative
F	45			41/91	246	Negative
M	41			37/87	245	Negative
M	36			36/88	237	Negative

a The AAM group included individuals vaccinated twice with AstraZeneca and once with Moderna vaccine; the AMM group included individuals vaccinated once with AstraZeneca and twice with Moderna vaccine; the MMM group included individuals vaccinated thrice with Moderna vaccine.

### 3.2 Decline in RBD-specific antibody titers across SARS-CoV-2 variants in vaccinated individuals

**Figure 1:**
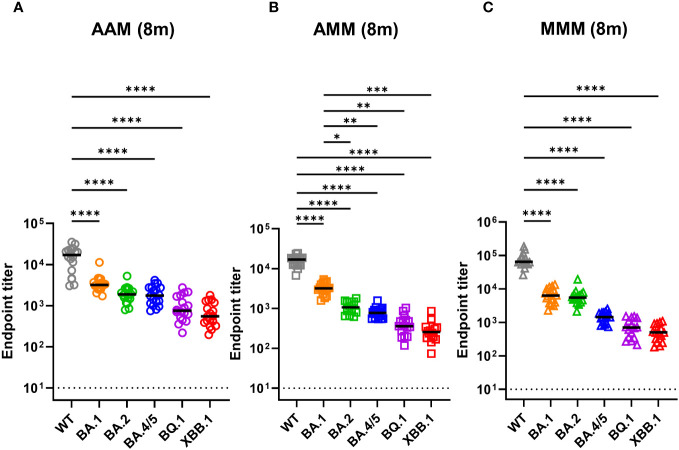
Decreases in RBD-recognizing antibodies against Omicron variants across all vaccination groups. **(A–C)** Measurement of anti-RBD antibody titers against ancestral spike WT (grey), Omicron variants BA.1 (orange), BA.2 (green), and BA.4/5 (blue), BQ.1 (purple), and XBB.1 (red) by indirect ELISA using serum from various vaccine combination groups: AAM (2X AstraZeneca + 1X Moderna) **(A)**, AMM (1X AstraZeneca + 2X Moderna) **(B)**, and MMM (3X Moderna) **(C)**. Blood samples were collected eight months post-vaccination after the final vaccine dose (8m). AAM (n = 18), AMM (n = 16), and MMM (n = 16). Duplicates were performed for each tested sample. Statistical significance was calculated among experiments by one-way ANOVA with a Tukey’s post-hoc test for multiple pairwise comparisons. The dotted line represents the cut-off value for each assay. Asterisks indicate statistical significance, *p.adj ≤ 0.05, **p.adj ≤ 0.01, ***p.adj ≤ 0.001, **** p.adj < 0.0001.

Inducing antibodies targeting the receptor-binding domain (RBD) of the SARS-CoV-2 spike protein is crucial for blocking virus infection. Thus, we determined the RBD-specific IgG titers of individuals with different vaccination schedules against different SARS-CoV-2 variants using ELISA (**Figure 1**). In the AAM group, the mean endpoint titers against WT, BA.1, BA.2, BA.4/5, BQ.1, and XBB.1 were 16,353, 3,630, 2,004, 2,011, 1,051, and 729.8 respectively (**Figure 1A**). Similarly, in the AMM group, the mean endpoint titers against WT, BA.1, BA.2, BA.4/5, BQ.1, and XBB.1 were 15,773, 3,304, 1,123, 804.8, 427.7, and 304.5 respectively (**Figure 1B**). The MMM group exhibited the highest antibody titers against the WT spike, with mean endpoint titers of 78,352, 6,983, 6,359, 1,490, 781.2, and 545.1 against WT, BA.1, BA.2, BA.4/5, BQ.1, and XBB.1, respectively (**Figure 1C**). Overall, across all groups, we observed a decline in RBD-specific IgG titers against the BA.1, BA.2, BA.4/5, BQ.1, and XBB.1 variants compared to the WT (Tukey’s multiple comparisons test p.adj <0.0001). When comparing the vaccine regimens, the MMM group had the highest antibody titer against the WT, BA.1, and BA.2 variants compared to the AAM or AMM group. However, when confronted with the BQ.1 and XBB.1 variants, the MMM group did not display a comparable antibody titer to the AAM or AMM group, suggesting that the antibodies induced by the WT vaccine may lose their ability to effectively recognize the Omicron BQ.1 and XBB.1 spike proteins, which harbors a significant number of mutations in the RBD.

### 3.3 Reduction in neutralizing activity of vaccinated individuals’ sera against SARS-CoV-2 variants

**Figure 2:**
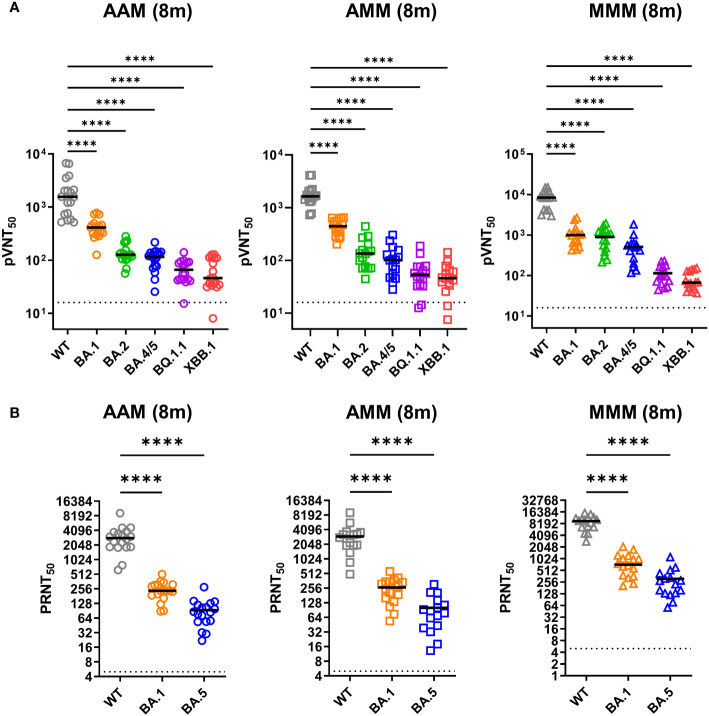
Robust resistance of Omicron variants to neutralization induced by diverse vaccination combinations. **(A, B)** Examination of pseudovirus neutralization (pVNT50) **(A)** and infectious SARS-CoV-2 neutralization (PRNT50) **(B)** against ancestral spike WT (grey), Omicron variants BA.1 (orange), BA.2 (green), BA.4/5 (blue), BQ.1.1 (purple), and XBB.1 (red) **(A)**, and WT, Omicron BA.1, and BA.5 **(B)**. The neutralization tests were conducted using sera from the AAM (n = 18), AMM (n = 16), and MMM (n = 16) vaccination groups, as previously described in **Figure 1**. Duplicates were performed for each tested serum. Statistical significance was calculated among experiments by one-way ANOVA with Tukey’s multiple comparison test. The dotted line represents the cut-off value for each assay. Asterisks indicate statistical significance, ****p.adj < 0.0001.

Next, we measured neutralizing titers against the emerging variants by using the pseudovirus and infectious virus neutralization assay (**Figure 2**). The AAM group showed the median of 50% pseudovirus neutralization titer (pVNT50) values of 2,104 (WT), 424.6 (BA.1), 141.7 (BA.2), 109.2 (BA.4/5), 66.18 (BQ.1.1), 66.12 (XBB.1). Similarly, the AMM group exhibited mean pVNT50 values of 1,867 (WT), 448.9 (BA.1), 154.5 (BA.2), 111.8 (BA.4/5), 62.53 (BQ.1.1), and 52.18 (XBB.1), and the MMM group had mean pVNT50 values of 8,330 (WT), 1,064 (BA.1), 897.4 (BA.2), 531.0 (BA.4/5), 114.1 (BQ.1.1), and 80.60 (XBB.1) (**Figure 2A**). Significant differences in neutralizing activity were observed when comparing the WT-induced polyvalent sera against the tested variants (WT, BA.1, BA.2, BA.4/5, BQ.1.1 and XBB.1) across different vaccination combinations (AAM, AMM, and MMM group), with the most substantial reduction in neutralizing antibody titers against the XBB.1 variant in the AAM, AMM, and MMM group, with a mean fold decrease of 31.8, 35.8 and 103, respectively (**Figure 2A**). Besides, the MMM group had the best neutralizing effect against the WT, BA.1, BA.2 and BA.4/5 compared to the AAM or AMM group (**Supplementary Figures 1A-D**). However, when faced with the BQ.1.1 and XBB.1 variants, which has accumulated a vast number of mutations in the RBD region, the neutralization titers are significantly reduced, regardless of the vaccination schedules (**Supplementary Figures 1E, F**).

In addition, we further evaluated the neutralizing activity by using the infectious virus neutralization assay (**Figure 2B**). The mean 50% plaque reduction neutralization test (PRNT50) values for the AAM group were 3,057 for the WT strain, 247.2 for the BA.1, and 93.6 for the BA.5 variants. Similarly, for the AMM group, the mean PRNT50 values were 3,041 for WT, 259.5 for BA.1 and 100.7 for BA.5. The MMM group exhibited mean PRNT50 values of 9,086 for WT, 847.5 for BA.1 and 310.5 for BA.5, showing the best neutralizing effect against the WT, BA.1 and BA.5 compared to the AAM or AMM group (**Figure 2B**). A significant reduction in the mean PRNT50 values for the BA.1 and BA.5 variant compared to WT was observed across different vaccine combinations. The AAM, AMM, and MMM groups showed approximately 12-fold, 11-fold, and 10.5-fold reductions for BA.1, and 32-fold, 30-fold, and 29.3-fold reductions for BA.5, respectively, in neutralizing activity compared to WT (**Figure 2B**). The detailed pVNT50 and PRNT50 values were summarized in the **Supplementary Table 1**. Overall, our findings suggest that the Omicron variants may possess specific mutations or epitope changes that affect the recognition and binding of neutralizing antibodies induced by the evaluated vaccination regimens.

### 3.4 Cellular immune responses to WT and Omicron variants

**Figure 3:**
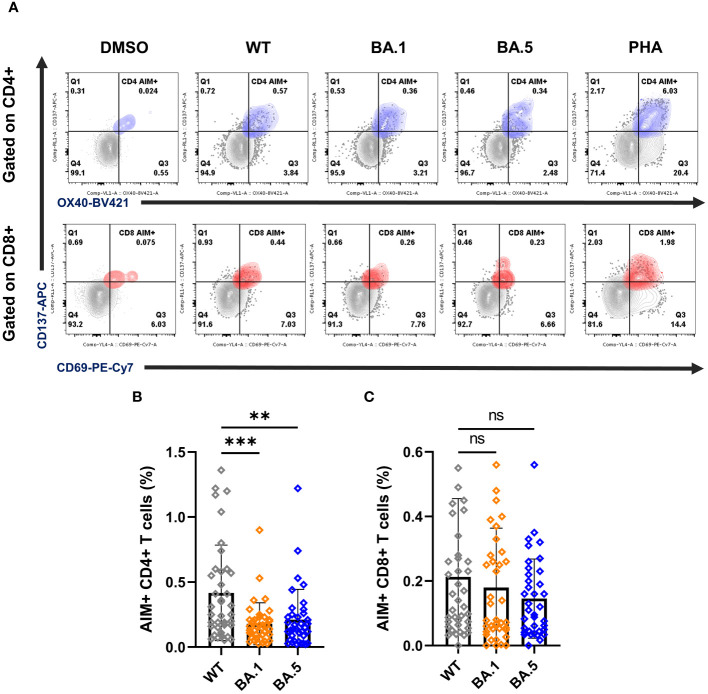
T cell responses of vaccinees against ancestral spike WT and variants BA.1 and BA.5. **(A)** Representative FLOW dot plots for spike-specific CD4+ and CD8+ T cells by the expression of OX40+CD137+ (blue) and CD69+CD137+ (red), respectively. PBMCs from vaccinees were stimulated with the peptide pools ancestral spike WT, the variants BA.1 and BA.5. DMSO and PHA were used as negative and positive controls, respectively. The gating strategy for the AIM assay is illustrated by representative graphs (**Supplementary Figure 3A**). **(B, C)** Percentages of AIM+ CD4+ (OX40+CD137+) **(B)** and AIM+ CD8+ (CD69+CD137+) T cells **(C)**. Samples PBMCs from all 38 vaccinees including AAM (n = 16), AMM (n = 12), and MMM (n = 10) were analyzed together here. Statistical significance was calculated among experiments by one-way ANOVA with a Tukey’s *post-hoc* test for multiple pairwise comparisons. Asterisks indicate statistical significance, **p.adj ≤ 0.01, ***p.adj ≤ 0.001. ns, not significant.

**Figure 4:**
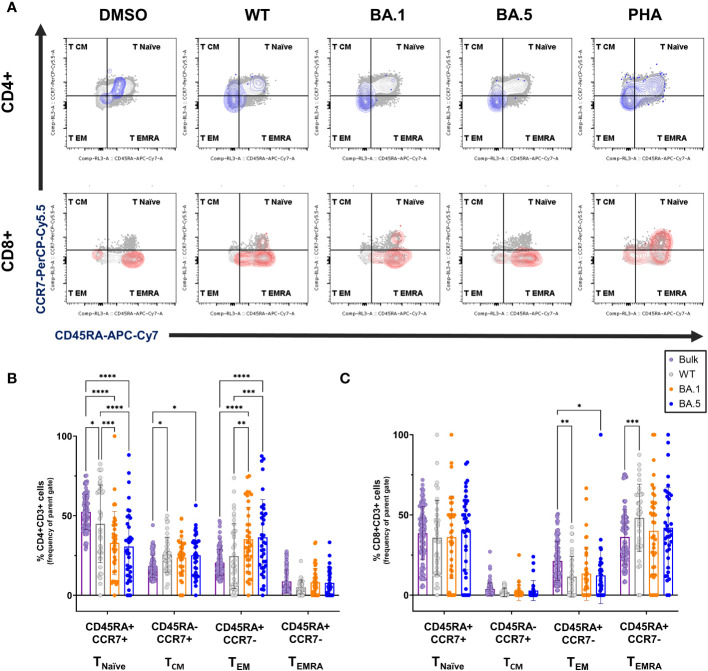
Memory phenotypes of the spike-specific T cells from vaccine recipients against ancestral spike WT and Omicron variants. **(A)** Representative FLOW gating plots for memory subsets naïve (CD45RA+CCR7+), central memory (CD45RA-CCR7+), effector memory (CD45RA-CCR7-), and terminally differentiated effector memory (CD45RA+CCR7-) on the bulk CD4+ and CD8+ T cells and the AIM+ subsets (blue for CD4+ and red for CD8+, respectively), responding to the ancestral spike WT, Omicron variants BA.1 and BA.5 peptide pools stimulation. **(B, C)** Frequencies of memory subsets T_Naïve_, T_CM_, T_EM_, and T_EMRA_ in bulk (purple) and AIM+ subsets induced by SARS-CoV-2 ancestral spike WT (grey), variants BA.1 (orange), or BA.5 (blue) on CD4+ **(B)** or CD8+ **(C)** T cells from vaccine recipients (n=38). Statistical significance was calculated among experiments by 2way ANOVA with a Tukey’s *post-hoc* test for multiple pairwise comparisons. Asterisks indicate statistical significance, *p.adj ≤ 0.05, **p.adj ≤ 0.01, ***p.adj ≤ 0.001, **** p.adj < 0.0001.

Cellular immune responses, including CD4+ and CD8+ T cell responses, play a crucial role in protecting against different SARS-CoV-2 variants infection. Thus, we next assessed spike-specific cellular immune responses using the AIM (activation-induced markers) assay (**Figure 3**; **Supplementary Figures 3**, **4**) and analyzed the memory phenotype within SARS-CoV-2-reactive AIM+ CD4 and CD8 T cells (**Figure 4**; **Supplementary Figure 5**). The PBMCs from 38 vaccinated individuals (16 from AAM, 12 from AMM, and 10 from MMM) within the participants from previous results were involved in the following experiments, allowing for simultaneous tracing of both humoral and cellular responses in the vaccinated individuals.

Flow cytometry was used to detect AIM expression on CD4+ (OX40+CD137+) and CD8+ (CD69+CD137+) T cells (**Figure 3A**; **Supplementary Figure 3A**). The mean percentages of AIM+ CD4+ T cells stimulated with spike WT, BA.1, and BA.5 peptide pools were 0.42%, 0.18%, and 0.21%, respectively, while the mean percentages of AIM+ CD8+ T cells under peptide stimulation were 0.21%, 0.18%, and 0.15% (**Figures 3B, C**). These results indicated that compared to WT, CD4 T cell reactivity against BA.1 and BA.5 showed significant reductions, with a 2.3-fold and 1.9-fold decrease, respectively. Besides, we observed a similar reduction in the AAM group when comparing the Omicron variants to the WT, not only in AIM expression on CD4+ T cells but also in the secretion of IFN-γ and IL-2 detected by ELISPOT assays (**Supplementary Figures 2**, **3**). Unlike the results from the AIM+ CD4+ assay, there was no significant difference detected in the mean percentages of AIM+ CD8+ T cells among the WT, BA.1, and BA.5 spike treatment groups (**Figure 3C**), suggesting that CD8+ T cells showed considerable cross-reactivity to Omicron even 8 months after vaccination. Moreover, we found that the population of AIM+ CD8+ T cells remained consistently higher in the MMM group than in the AAM group, whereas similar results could not be found in AIM+ CD4+ T cells (**Supplementary Figure 4**). The detailed T cell reactivities were summarized in the **Supplementary Table 2**.

We further explored memory phenotypes within AIM+ CD4+ and CD8+ T cell subsets among vaccinated individuals with diverse regimens, including naïve T cells (T_Naïve_), central memory T cells (T_CM_), effector memory T cells (T_EM_), and CD45RA+ effector memory T cells (T_EMRA_) in response to WT, BA.1 or BA.5 variants (**Figure 4** and **Supplementary Figure 5**). 8 months after receiving three vaccine doses, the CD4+ spike-specific memory T cells were primarily T_CM_ and T_EM_, while CD8+ subsets were mainly T_EM_ and T_EMRA_, excluding naive cells. In CD4+ T cells, we noted a decline in the naïve population and a preferential enrichment in the T_CM_ and T_EM_ populations compared to the bulk counterpart (**Figures 4A, B**). Conversely, for CD8+ T cells, the difference was less pronounced. However, WT spike-specific AIM+ CD8+ T cells exhibited significantly higher proportions of T_EMRA_ and lower T_EM_ percentages than the bulk population (**Figures 4A, C**). When focusing on distinctions between the variants, we noted a rise in the population of T_EM_ cells in response to Omicron variants compared to WT, coupled with a decrease in T_Naïve_ cells. The detailed memory phenotypes of T cell subsets were summarized in the **Supplementary Table 3**.

Across varying vaccine combinations, memory subsets showed consistent trends: diminished naïve T cells and increased effector memory in CD4+ T cells (**Supplementary Figure 5**, left panels), with fewer differences observed in CD8+ T cells between original WT and variants (**Supplementary Figure 5**, right panels). In summary, these findings offer additional insights into T cell memory phenotypes in vaccinated individuals primed by the ancestral WT spike protein, revealing unique subsets that may contribute to long-term cross-reactive immunity against Omicron variants.

## 4 Discussion

Despite the availability of vaccines, the pandemic has not been fully controlled, and infections are as high as ever through Omicron. Thus, in this study, we examined a detailed analysis of the humoral and cellular immunities for up to 8 months against SARS-CoV-2 WT and Omicron variants after different triple vaccinations in the Taiwanese population.

Based on our ELISA (**Figure 1**) and pseudovirus neutralization assays (**Figure 2A**), a significant wane in RBD-specific IgG titers and pVNT50 values against Omicron variants, such as BA.1, BA.4/5, BQ.1.1 and XBB.1, were observed in all participants compared to WT. Moreover, the PRNT50 values of Omicron variants BA.1 and BA.5 were also significantly lower than WT upon infectious virus neutralization assays (**Figure 2B**). The evasion of humoral immune responses may be attributed to the significant changes in the Spike protein of Omicron variants compared with WT (24, 25). These results may raise some concerns about the long-term protection of future emerging variants of SARS-CoV-2.

In addition to the neutralizing activity, we also found that the AIM+ CD4+ responses decline rapidly from WT to omicron variants (**Figure 3B**). However, the AIM+ CD8+ population (**Figure 3C**) and memory subsets of CD4+ and CD8+ T cells (**Figure 4**) responding to WT and the variants BA.1, and BA.5 lineages remained largely unaffected. Furthermore, we discovered an increase in SARS-CoV-2-specific T_CM_ and T_EM_ in CD4 T cells (**Figure 4B**), as well as T_EMRA_ subsets in CD8 T cells (**Figure 4C**), both T_EM_ and T_EMRA_ cells play critical roles in antiviral immunity (26, 27). Previous studies showed that transcriptional signature in long-lived memory CD8+ T cells after acute SARS-CoV-2 infection supports the notion that inducing T_EMRA_ in response to variant spike stimulation may sustain long-term protection despite ongoing variant emergence (28, 29). Some studies revealed that T_EM_ and T_CM_ subsets of SARS-CoV-2–specific CD4+ T cells were induced after the infection, and the population can remain over half a year (30, 31). Notably, we observed an increase in the proportion of Omicron-specific T_EM_ cells in CD4 T cells compared to the proportion against the WT (**Figure 4B**; **Supplementary Figure 5**, left panels). The results showed that, despite the significant drop in AIM+ CD4+ T cells against Omicron variants (**Figure 3B**), the larger proportion of effector memory cells may still offer sustainable protection for up to 8 months. (**Figure 4B**). These results offer some evidence that overall T cell responses are not significantly disrupted by the Omicron variants BA.1 and BA.5. However, it remains intriguing to monitor cellular immunity against evolving Omicron variants, such as BA.2, BQ.1.1 or XBB.1, in individuals who have received triple vaccines without infection. Unfortunately, this experiment is currently inaccessible, as a large number of PBMCs from study participants were utilized in the **Figures 3** and **4**, we don’t have enough PBMCs to perform with the remaining variants such as BA.2, BQ.1.1 or XBB.1, etc. Moreover, since Taiwan has been open for a while with few infection-free cases, difficulties have arisen in recruiting additional participants in this study.

Previous studies have reported that mRNA-based vaccines induce higher neutralization abilities (32, 33) and CD8+ T cell responses (34, 35) than vector-based vaccines after one or two vaccination doses. However, it remains to be evaluated how well-vaccine-induced immunity is preserved over time after receiving three doses of vaccines, especially for the circulating Omicron subvariants BQ.1.1 and XBB.1. In our study, we found higher neutralizing activity in the MMM group compared to the AAM group across all variants based on pseudovirus and infectious virus neutralization assays (**Figure 2** and **Supplementary Figure 1**). Furthermore, we observed a similar trend in cellular responses, with the population of AIM+ CD8+ T cells consistently higher in the MMM group (**Supplementary Figures 4D-F**). Therefore, participants receiving three doses of mRNA-based vaccines might have better protection against SARS-CoV-2 variants even after 8 months. These findings have implications for vaccine-induced immunity and provide insight into potential vaccine strategies.

However, our study has limitations, including small sample sizes obtained through convenience sampling. Additionally, the age range of our study cohort (27 to 63 years) limits the generalization of the immunogenicity results to children, older populations, and individuals of non-Asian descent. We also used overlapping peptide pools to stimulate PBMCs. While minor amino acid changes within peptide sequences can affect T cell receptor (TCR) recognition and major histocompatibility complex (MHC) binding, we could not pinpoint the specific changes primarily influencing variant-related T cell responses. Moreover, our cohorts were predominantly Taiwanese individuals, reflecting our recruitment pool. Notably, MHC allele prevalence varies across regions, and the preference of specific MHC-I or MHC-II molecules for presenting foreign peptides impacts immune responses to foreign antigens. Given these cohort constraints, we may not generalize memory T cell subsets’ cross-reactivity to Omicron variants to regions where individuals may express different dominant MHC alleles.

## 5 Conclusion

Our findings provide an encompassing view of SARS-CoV-2 immunity against WT and evolving Omicron variants. Although the sustained CD8+ T cell responses and the induction of unique memory T cell subsets by Omicron spike protein stimulation demonstrate potential for enduring protection, a significant reduction in the humoral and CD4+ T cell responses against Omicron variants implies that long-term protection may not be sufficient to protect against the reinfection of emerging variants. Thus, receiving another booster dose or getting inoculated with BA.4/5- or XBB.1-containing vaccines may still be the most effective approach to dealing with the virus and the ongoing pandemic.

## Data availability statement

The original contributions presented in the study are included in the article/**Supplementary Material**. Further inquiries can be directed to the corresponding author.

## Ethics statement

The studies involving humans were approved by Institutional Review Board of Tri-Service General Hospital (TSGHIRB No. B202105097). The studies were conducted in accordance with the local legislation and institutional requirements. The participants provided their written informed consent to participate in this study. Ethical approval was not required for the studies on animals in accordance with the local legislation and institutional requirements because only commercially available established cell lines were used.

## Author contributions

CH: Investigation, Writing – review & editing, Writing – original draft, Visualization, Resources, Methodology, Formal analysis, Data curation, Conceptualization. LY: Writing – review & editing, Writing – original draft, Visualization, Validation, Resources, Methodology, Investigation, Funding acquisition, Formal analysis, Data curation, Conceptualization. HH: Writing – review & editing, Writing – original draft, Resources, Methodology, Investigation, Formal analysis, Data curation, Conceptualization. CL: Writing – review & editing, Investigation, Funding acquisition, Data curation, Conceptualization. YH: Writing – review & editing, Visualization, Validation, Software, Data curation. CL: Writing – review & editing, Investigation, Conceptualization, Visualization, Validation, Software, Resources. CH: Writing – review & editing, Resources, Methodology, Formal analysis. KC: Writing – review & editing, Writing – original draft, Visualization, Validation, Supervision, Resources, Project administration, Methodology, Investigation, Funding acquisition, Formal analysis, Data curation, Conceptualization.
