# Supplementary material for: Long-term analysis of humoral responses and spike-specific T cell memory to Omicron variants after different COVID-19 vaccine regimens
Source: Front Immunol. 2024 Mar 12;15:1340645. doi: 10.3389/fimmu.2024.1340645 (PMC10963495; doi:10.3389/fimmu.2024.1340645)
Supplement: Supplementary file 1 [file Image_1.pdf]

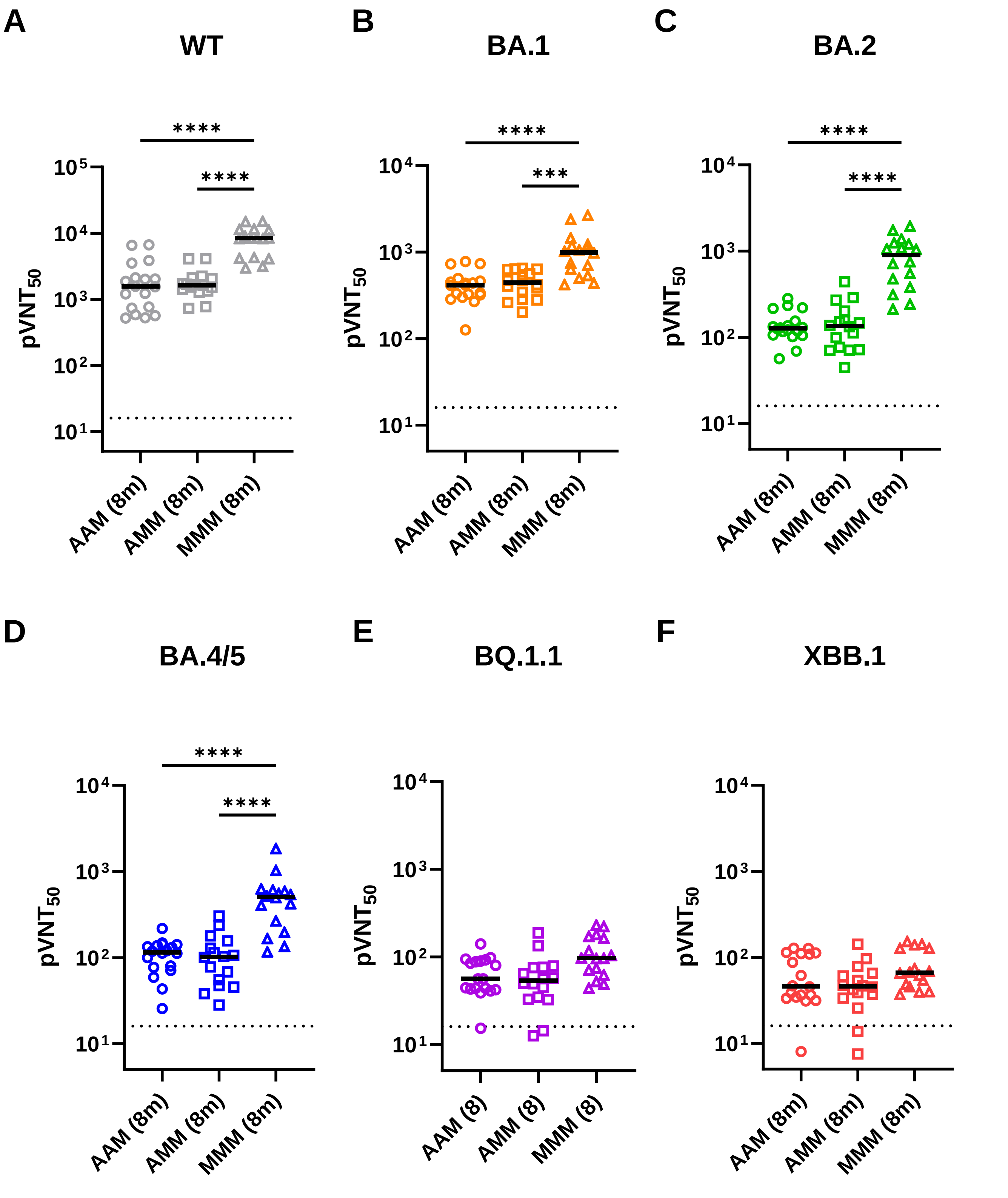

Supplementary Figure 1

Robust resistance of Omicron variants to neutralization across diverse vaccine combinations. **(A-F)** Analysis of pseudovirus neutralization (pVNT50) against ancestral spike WT (grey) **(A)**, Omicron variants BA.1 (orange) **(B)**, BA.2 (green) **(C)**, BA.4/5 (blue) **(D)**, BQ.1.1 (purple) **(E)**, and XBB.1 (red) **(F)**, respectively, utilizing the same dataset as presented in Figure 2A. Duplicates were performed for each tested serum. Statistical significance was calculated among experiments by one-way ANOVA with Tukey's multiple comparison test. The dotted line represents the cut-off value for each assay. Asterisks indicate statistical significance, ****p.adj < 0.0001.
