# Supplementary material for: Long-term analysis of humoral responses and spike-specific T cell memory to Omicron variants after different COVID-19 vaccine regimens
Source: Front Immunol. 2024 Mar 12;15:1340645. doi: 10.3389/fimmu.2024.1340645 (PMC10963495; doi:10.3389/fimmu.2024.1340645)
Supplement: Supplementary file 2 [file Image_2.pdf]

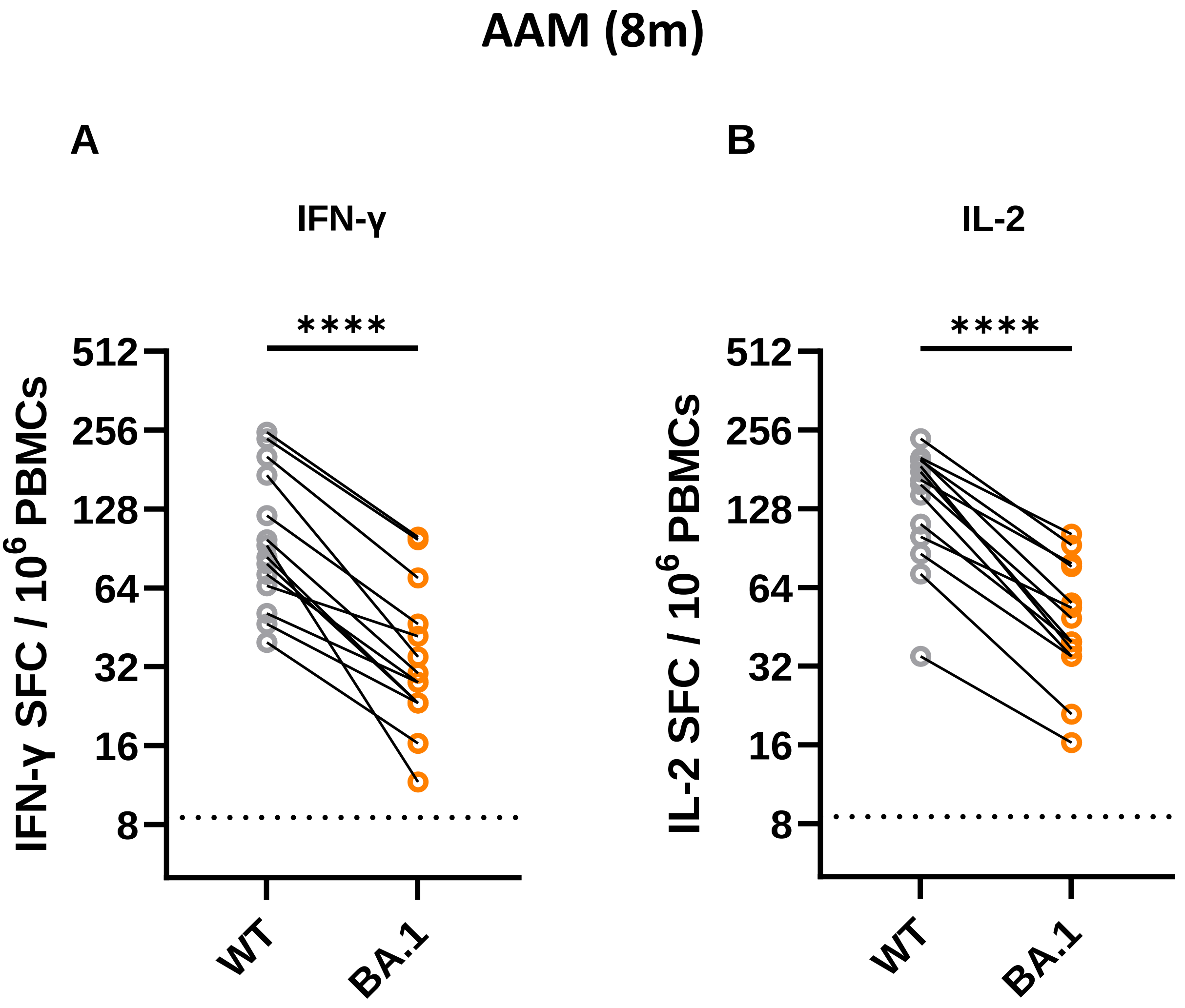

Supplementary Figure 2

Comparison of cellular immune response changes between WT and BA.1 spike peptide stimulation. **(A-B)** Detection of IFN-γ-secreting T cells **(A)** and IL-2-secreting T cells **(B)** in the AAM vaccine regimen group. Duplicates were performed for each tested sample. Measured statistical significance was calculated among SARS-CoV-2 variants by two-tailed Student's t test. Asterisks indicate statistical significance, ****$p < 0.0001$.
