# Supplementary material for: Long-term analysis of humoral responses and spike-specific T cell memory to Omicron variants after different COVID-19 vaccine regimens
Source: Front Immunol. 2024 Mar 12;15:1340645. doi: 10.3389/fimmu.2024.1340645 (PMC10963495; doi:10.3389/fimmu.2024.1340645)
Supplement: Supplementary file 3 [file Image_3.pdf]

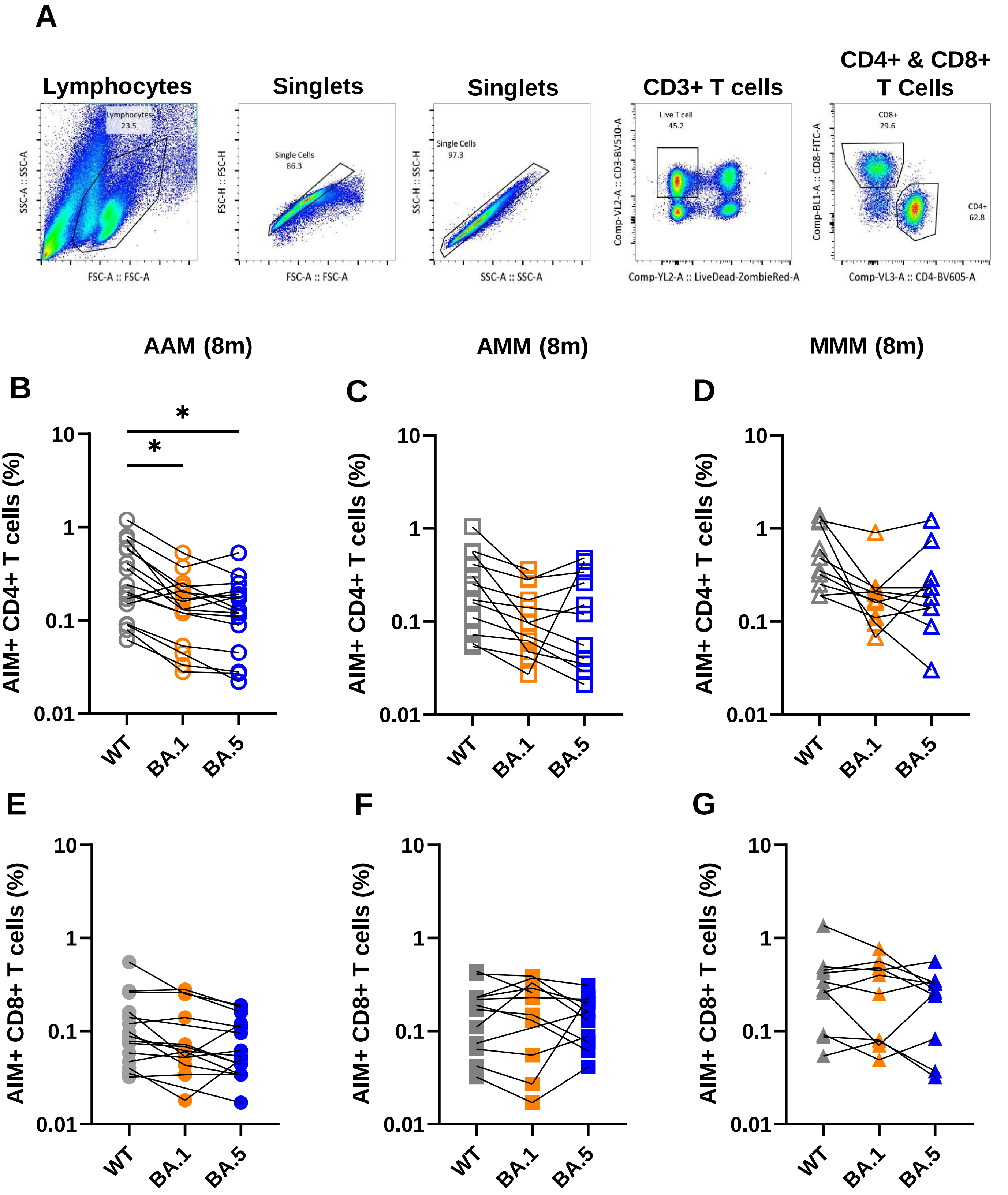

Supplementary Figure 3

T cell responses of vaccinees against ancestral spike WT and variants BA.1 and BA.5 in specific vaccination groups. **(A)** Gating strategy for the AIM assay, relevant to Figure 3. **(B-G)** Detailed examination of the decreases in AIM percentages in CD4+ **(B-D)** or CD8+ **(E-G)** T cells within the specific vaccination groups AAM, AMM, or MMM, utilizing the same dataset as presented in Figure 3. Statistical significance was calculated among experiments by one-way ANOVA with a Tukey's post-hoc test for multiple pairwise comparisons. Asterisks indicate statistical significance, *p.adj ≤ 0.05.
