# Supplementary material for: Long-term analysis of humoral responses and spike-specific T cell memory to Omicron variants after different COVID-19 vaccine regimens
Source: Front Immunol. 2024 Mar 12;15:1340645. doi: 10.3389/fimmu.2024.1340645 (PMC10963495; doi:10.3389/fimmu.2024.1340645)
Supplement: Supplementary file 4 [file Image_4.pdf]

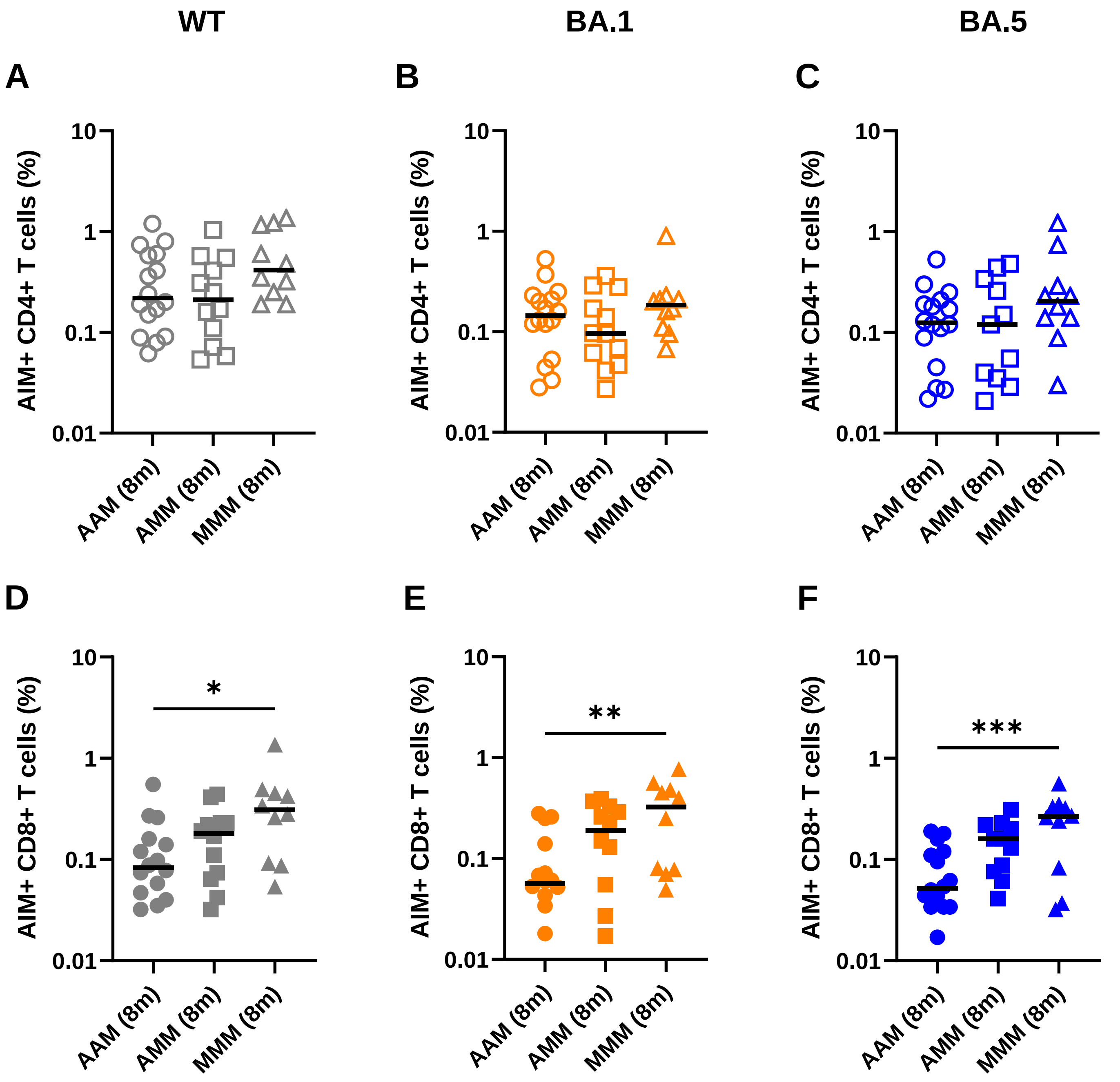

Supplementary Figure 4

T cell responses of vaccinees against specific SARS-CoV-2 spike variants across diverse vaccine combinations. **(A-F)** Percentages of AIM+ CD4+ (OX40+CD137+) **(A-C)** and AIM+ CD8+ (CD69+CD137+) T cells **(D-F)** against ancestral spike WT (grey), variants BA.1 (orange), and BA.5 (blue), utilizing the same dataset as presented in Figure 3. Statistical significance was calculated among experiments by one-way ANOVA with a Tukey's post-hoc test for multiple pairwise comparisons. Asterisks indicate statistical significance, *p.adj $\leq$ 0.05, **p.adj $\leq$ 0.01, ***p.adj $\leq$ 0.001.
