# Supplementary material for: Long-term analysis of humoral responses and spike-specific T cell memory to Omicron variants after different COVID-19 vaccine regimens
Source: Front Immunol. 2024 Mar 12;15:1340645. doi: 10.3389/fimmu.2024.1340645 (PMC10963495; doi:10.3389/fimmu.2024.1340645)
Supplement: Supplementary file 5 [file Image_5.pdf]

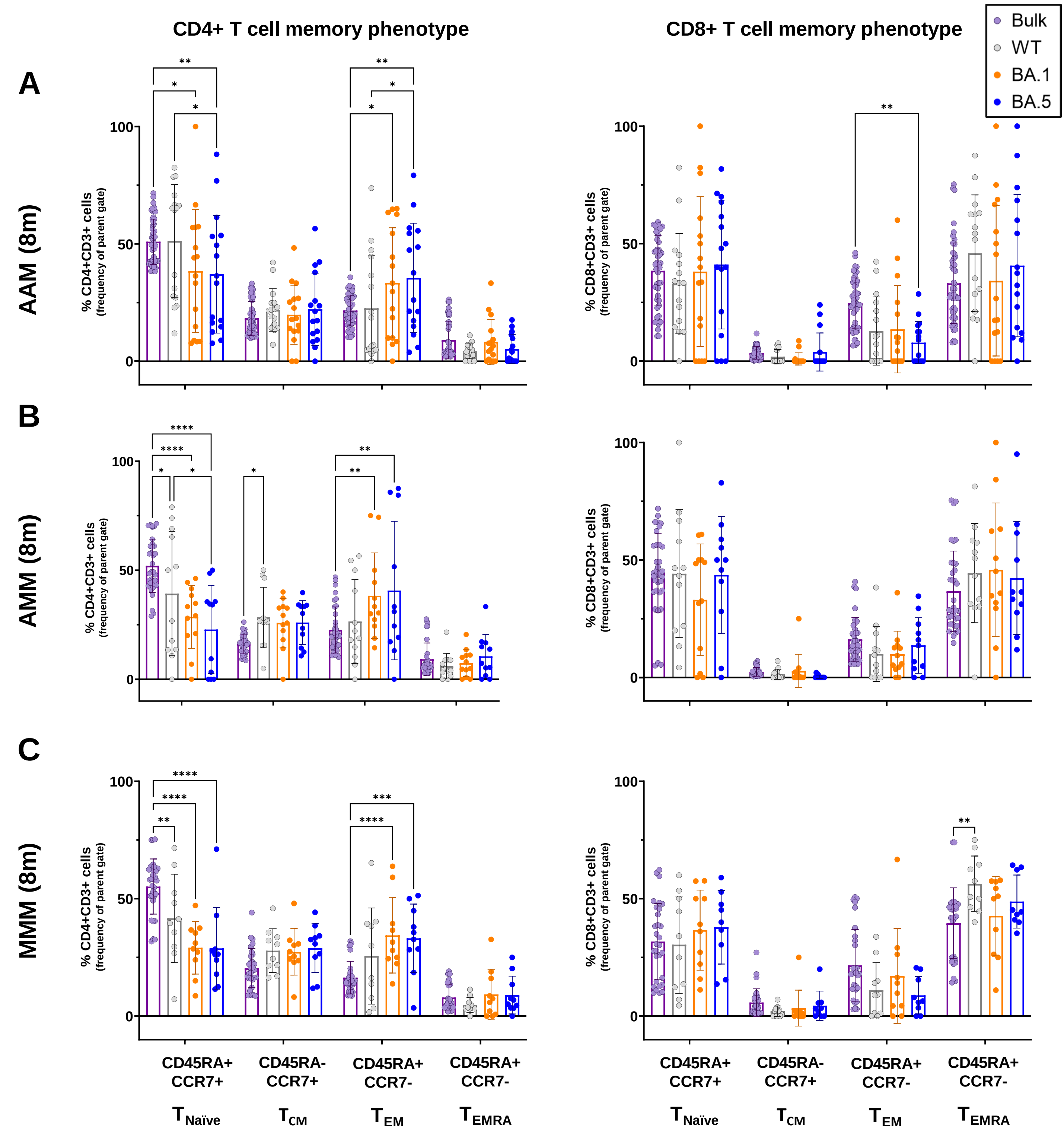

Supplementary Figure 5

Memory phenotypes of the spike-specific T cells from vaccine recipients in specific vaccination groups. FLOW gating strategy for memory subsets naïve (CD45RA+CCR7+), central memory (CD45RA-CCR7+), effector memory (CD45RA-CCR7-), and terminally differentiated effector memory (CD45RA+CCR7-) as presented in Figure 4A. **(A-C)** Frequencies of memory subsets $T_{Naïve}$, T_{CM}, T_{EM}, and T_{EMRA} in bulk (purple) and AIM+ subsets induced by SARS-CoV-2 ancestral spike WT (grey), variants BA.1 (orange), or BA.5 (blue) on CD4+ (left) or CD8+ (right) T cells from specific vaccination groups AAM (n = 16) **(A)**, AMM (n = 12) **(B)**, and MMM (n = 10) **(C)**, utilizing the same dataset as presented in Figure 4. Statistical significance was calculated among experiments by 2way ANOVA with a Tukey's post-hoc test for multiple pairwise comparisons. Asterisks indicate statistical significance, *p.adj ≤ 0.05, **p.adj ≤ 0.01, ***p.adj ≤ 0.001, ****p.adj < 0.0001.
