# Supplementary material for: Long-term analysis of humoral responses and spike-specific T cell memory to Omicron variants after different COVID-19 vaccine regimens
Source: Front Immunol. 2024 Mar 12;15:1340645. doi: 10.3389/fimmu.2024.1340645 (PMC10963495; doi:10.3389/fimmu.2024.1340645)
Supplement: Supplementary file 6 [file Table_1.docx]

**Supplementary Table 1- Neutralization potency of each participant against pseudovirus and infectious virus**

| **Gender** | **Age** | **Underlying disease** | **Vaccination schedule** | **pVNT_50_ (AAM, AMM, MMM)** | | | | | | **PRNT_50_** | | |
| --- | --- | --- | --- | --- | --- | --- | --- | --- | --- | --- | --- | --- |
|  |  |  |  | **WT** | **BA.1** | **BA.2** | **BA.4/5** | **BQ.1.1** | **XBB.1** | **WT** | **BA.1** | **BA.5** |
| M | 54 | Hypertension | AAM | 522.1 | 267.7 | 105.1 | 25.5 | 40.9 | 36.4 | 783.1 | 187.4 | 56.2 |
| F | 27 |  |  | 1205.8 | 734.6 | 68.9 | 43.3 | 88.1 | 87.1 | 1808.6 | 514.2 | 277.7 |
| M | 38 |  |  | 2032 | 450 | 130.5 | 79.7 | 97.6 | 127.9 | 3048 | 315 | 72.4 |
| F | 56 | Hyperlipidemia |  | 1224.9 | 495.3 | 219.7 | 111.5 | 92.2 | 113.7 | 1837.4 | 346.7 | 84.3 |
| M | 46 |  |  | 518.5 | 286.2 | 56.3 | 58.9 | 42 | 38.6 | 622.2 | 200.3 | 86.3 |
| F | 53 |  |  | 2014 | 439.3 | 101.7 | 118.3 | 43.7 | 34.5 | 2416.8 | 307.5 | 98.4 |
| M | 58 | Hypertension |  | 2109.1 | 777.8 | 128.5 | 119.5 | 84.8 | 61.5 | 4644.3 | 233.3 | 53.9 |
| F | 45 |  |  | 1542.9 | 413.7 | 118 | 133.6 | 89.4 | 110.1 | 2291.4 | 124.1 | 53.7 |
| M | 41 |  |  | 3870.3 | 300.8 | 232.9 | 130.8 | 15.3 | 8 | 3227.5 | 90.2 | 22 |
| M | 34 |  |  | 6668.6 | 320.1 | 281.5 | 216.7 | 43.7 | 36.5 | 3853.1 | 224.1 | 30.1 |
| F  F  M  M  F  M  F  M | 32  35  51  42  37  46  31  56 | Hyperlipidemia  Hypertension |  | 1571.3  1868  765.3  584.6  562.1  3531.3  6545.4  734.6 | 412.2  126.1  335.9  331.3  436.1  326.3  462.1  727.5 | 116.1  154.5  135.8  132.4  124.5  106.3  122  215.7 | 76.8  141  99.6  70.7  137.5  112.3  147.3  142.9 | 38.9  43  56  94.4  140.7  80.1  56  44.4 | 33.3 | 3006 | 288.5 | 77 |
|  |  |  |  |  |  |  |  |  | 31 | 2688.8 | 88.3 | 32.3 |
|  |  |  |  |  |  |  |  |  | 46.6 | 1865.9 | 235.1 | 119.9 |
|  |  |  |  |  |  |  |  |  | 108.9 | 1869.2 | 231.9 | 139.2 |
|  |  |  |  |  |  |  |  |  | 112.6 | 9223.3 | 305.3 | 104.4 |
|  |  |  |  |  |  |  |  |  | 126.5 | 4634 | 163.1 | 106.4 |
|  |  |  |  |  |  |  |  |  | 45.4 | 3763.1 | 231.1 | 143.3 |
|  |  |  |  |  |  |  |  |  | 31.4 | 3451.3 | 363.8 | 128 |
| M  M  F  F  M  F  M  F  F  M  F  F  M  F  M  M | 54  45  31  55  44  59  34  63  57  38  59  36  49  39  41  36 | Hypertension  Hypertension  Hyperlipidemia  Hypertension | AMM | 728.5  2063.1  4079.5  1286  1642.5  773.5  2108.1  4124.5  1331  1687.5  1500.7  1737.5  2233.1  1421.3  1525.3  1625.5 | 558.5  470.7  343.1  261.4  203.1  635  547.2  419.6  284.8  279.6  385.6  470.5  650.6  631.6  638.3  403.2 | 112.6  289.3  151.4  44.7  76.3  70.5  156.9  70.7  99  146.6  441.9  201.4  269.2  137.3  71.7  132.2 | 28.1  38.1  45.5  55.4  68.3  78  100.4  106.5  128.4  236.7  303.8  178.4  114.6  103.2  156.3  47.3 | 187.6  44.9  75.7  49.3  50.1  57.5  64.4  12.5  14.3  75.6  32.6  134.2  78.2  32.5  56.4  34.6 | 141.8 | 861.4 | 206.2 | 61.8 |
|  |  |  |  |  |  |  |  |  | 37 | 1989.5 | 565.6 | 305.4 |
|  |  |  |  |  |  |  |  |  | 78.6 | 5657.6 | 378 | 86.9 |
|  |  |  |  |  |  |  |  |  | 60.8 | 2204.9 | 416.1 | 101.1 |
|  |  |  |  |  |  |  |  |  | 38.9 | 497.8 | 160.3 | 69.1 |
|  |  |  |  |  |  |  |  |  | 42.1 | 1933.4 | 246 | 78.7 |
|  |  |  |  |  |  |  |  |  | 46.7 | 3715.5 | 186.7 | 43.1 |
|  |  |  |  |  |  |  |  |  | 13.7 | 1374.9 | 74.5 | 32.2 |
|  |  |  |  |  |  |  |  |  | 7.5 | 1936.5 | 54.1 | 13.1 |
|  |  |  |  |  |  |  |  |  | 54.1 | 3311.8 | 134.4 | 18 |
|  |  |  |  |  |  |  |  |  | 25.8 | 3607.2 | 346.2 | 92.4 |
|  |  |  |  |  |  |  |  |  | 96.8 | 3226.5 | 105.9 | 38.8 |
|  |  |  |  |  |  |  |  |  | 65.2 | 3239.1 | 282.1 | 143.9 |
|  |  |  |  |  |  |  |  |  | 33.6 | 2803.9 | 347.9 | 208.7 |
|  |  |  |  |  |  |  |  |  | 45.1 | 9145.6 | 335.8 | 114.8 |
|  |  |  |  |  |  |  |  |  | 47.2 | 3145.3 | 312.4 | 203.7 |
| M  F  M  F  M  F  F  M  F  M  M  F  M  F  M  M | 44  54  34  57  35  37  43  51  47  58  34  31  59  45  41  36 |  | MMM | 4096  14903.7  2971.7  8351.6  11371.5  4256  15063.7  3131.7  8511.6  11531.5  8951.6  8451.6  8221.4  11063.5  8267.7  4136.6 | 2382.1  1018.1  496.9  435.7  972.4  2654.1  1222.1  700.9  639.7  1176.4  1115.6  535.7  1456.2  421.2  742.4  1056.2 | 242.1  378.5  477.5  212.5  1950.2  553  1059.2  1207.5  1076.8  1378.7  714.6  312.5  1751.2  752.5  1043.2  1247.6 | 495.7  116.4  1024.1  165.4  1837.8  196.8  588.1  607.7  420.2  517.6  134  265.4  538.5  627.7  403.5  556.6 | 48.7  52.2  74.1  43.9  163.4  95.5  116.9  229.9  179.5  96.2  62.1  221.3  170.9  96.5  70.8  103.3 | 36.8 | 4542  10490.2  7069  10289.4  2862.1  11117.2  15506.1  4624  11941.8  14256.3  8637.2  6670.4  6621.3  11645.4  9368.9  9731.4 | 760.9  2087.7  1234.7  1759.2  645.1  990.3  686  364.9  233.7  580.3  1252.2  328.4  1029.8  1015.9  384.7  205.6 | 228.3 |
|  |  |  |  |  |  |  |  |  | 39.8 |  |  | 1127.4 |
|  |  |  |  |  |  |  |  |  | 54.4 |  |  | 284 |
|  |  | Hyperlipidemia |  |  |  |  |  |  | 39.5 |  |  | 427.5 |
|  |  |  |  |  |  |  |  |  | 126.7 |  |  | 278 |
|  |  |  |  |  |  |  |  |  | 65.1 |  |  | 316.9 |
|  |  |  |  |  |  |  |  |  | 74.1 |  |  | 158.5 |
|  |  |  |  |  |  |  |  |  | 152.1 |  |  | 158 |
|  |  |  |  |  |  |  |  |  | 127.2 |  |  | 56.9 |
|  |  | Hyperlipidemia |  |  |  |  |  |  | 68.8 |  |  | 77.9 |
|  |  |  |  |  |  |  |  |  | 45.4 |  |  | 334.3 |
|  |  |  |  |  |  |  |  |  | 139.5 |  |  | 120.2 |
|  |  | Hypertension |  |  |  |  |  |  | 142.5 |  |  | 525.2 |
|  |  |  |  |  |  |  |  |  | 61.6 |  |  | 609.5 |
|  |  |  |  |  |  |  |  |  | 48.8 |  |  | 131.6 |
|  |  |  |  |  |  |  |  |  | 67.4 |  |  | 134 |
