# Supplementary material for: Long-term analysis of humoral responses and spike-specific T cell memory to Omicron variants after different COVID-19 vaccine regimens
Source: Front Immunol. 2024 Mar 12;15:1340645. doi: 10.3389/fimmu.2024.1340645 (PMC10963495; doi:10.3389/fimmu.2024.1340645)
Supplement: Supplementary file 7 [file Table_2.docx]

**Supplementary Table 2- T cell reactivity of each participant against WT, BA.1, and BA.5**

| **Gender** | **Age** | **Underlying disease** | **Vaccination schedule** | **CD4+ AIM+ T cells (%)** | | | **CD8+ AIM+ T cells (%)** | | | **SFC / 10^6^ PBMCs** | | | |
| --- | --- | --- | --- | --- | --- | --- | --- | --- | --- | --- | --- | --- | --- |
|  |  |  |  | **WT** | **BA.1** | **BA.5** | **WT** | **BA.1** | **BA.5** | **WT peptide** | | **BA.1 peptide** | |
|  |  |  |  |  |  |  |  |  |  | **IFN-γ** | **IL-2** | **IFN-γ** | **IL-2** |
| F  M  F  M  F  M  F  M  M  F  F  M  M  F  M  F | 27  38  56  58  45  34  35  42  46  37  31  54  46  53  41  32 | Hyperlipidemia  Hyperlipidemia  Hypertension | AAM | 0.079  0.089  0.8  0.41  0.6  0.74  0.58  0.062  0.091  0.36  0.24  0.15  1.2  0.17  0.19  0.2 | 0.028  0.044  0.37  0.17  0.2  0.13  0.23  0.033  0.053  0.13  0.16  0.21  0.53  0.25  0.12  0.12 | 0.027  0.022  0.53  0.19  0.17  0.18  0.25  0.028  0.045  0.12  0.21  0.12  0.3  0.13  0.11  0.089 | 0.035  0  0.26  0.04  0.032  0.16  0.098  0.078  0.14  0.074  0.12  0.27  0.55  0.058  0.088  0.047 | 0  0.00322  0.26  0.018  0.034  0.053  0.043  0.072  0  0.068  0.14  0.28  0.25  0.052  0.061  0.06 | 0.017  0  0.16  0.05  0.034  0.12  0.034  0.044  0.095  0.044  0.11  0.18  0.19  0.062  0.054  0.034 | 98  172  72  121  65  79  202  X  93  51  251  47  X  237  40  84 | 35  158  100  177  198  186  112  X  72  86  200  195  X  165  144  237 | 30  35  28  47  X  42  23  70  12  28  X  100  23  98  16  23 | 16  49  53  40  X  56  37  40  21  35  X  102  77  79  35  93 |
| M  F  F  F  M  M  F  M  F  M  F  M | 45  31  59  57  38  36  55  44  55  34  36  49 | Hypertension  Hypertension | AMM | 0.31  0.054  0.058  0.41  1.04  0.57  0.25  0.072  0.11  0.16  0.17  0.55 | 0.047  0.041  0.027  0.28  0.29  0.36  0.17  0.062  0.069  0.097  0.14  0.096 | 0.035  0.021  0.44  0.48  0.34  X  0.26  0.029  0.04  0.15  0.12  0.055 | 0.032  0.074  0.19  0.064  0.042  0.44  0.23  0.11  0.23  0.22  0.41  0.17 | 0.017  0  0.13  0.055  0.027  0.26  0.37  0.33  0.29  0.23  0.39  0.15 | 0.041  0.16  0.061  0.088  0.23  X  0.31  0.13  0.2  0.22  0.16  0.076 |  | |  | |
| M  F  F  M  M  F  M  F  F  M | 41  43  54  44  34  57  35  37  47  51 | Hyperlipidemia | MMM | 1.22  1.36  0.35  0.6  0.48  0.25  0.32  0.19  1.17  0.19 | 0.9  0.21  0.2  0.095  0.23  0.17  0.16  0.21  0.067  0.11 | 1.22  0.74  0.14  0.03  0.23  0.088  0.23  0.18  0.29  0.14 | 0.49  0.092  0.45  1.36  0.34  0.42  0.26  0.087  0.054  0.28 | 0.45  0.049  0.56  0.77  0.25  0.48  0.4  0.08  0.078  0.07 | 0.56  0.083  0.33  0.27  0.35  0.24  0.32  0.032  0.037  0.26 |  | |  | |
