# Supplementary material for: Long-term analysis of humoral responses and spike-specific T cell memory to Omicron variants after different COVID-19 vaccine regimens
Source: Front Immunol. 2024 Mar 12;15:1340645. doi: 10.3389/fimmu.2024.1340645 (PMC10963495; doi:10.3389/fimmu.2024.1340645)
Supplement: Supplementary file 8 [file Table_3.docx]

**Supplementary Table 3- Memory phenotype of each participant**

| **Gender** | **Age** | **Underlying disease** | **Vaccination schedule** | **% CD4+CD3+ cells (frequency of parent gate)** | | | | | | | | | | | | **% CD8+CD3+ cells (frequency of parent gate)** | | | | | | | | | | | |
| --- | --- | --- | --- | --- | --- | --- | --- | --- | --- | --- | --- | --- | --- | --- | --- | --- | --- | --- | --- | --- | --- | --- | --- | --- | --- | --- | --- |
|  |  |  |  | **WT** | | | | **BA.1** | | | | **BA.5** | | | | **WT** | | | | **BA.1** | | | | **BA.5** | | | |
|  |  |  |  |  |  |  |  |  |  |  |  |  |  |  |  |  |  |  |  |  |  |  |  |  |  |  |  |
| F  M  F  M  F  M  F  M  M  F  F  M  M  F  M  F | 27  38  56  58  45  34  35  42  46  37  31  54  46  53  41  32 | Hyperlipidemia  Hypertension  Hypertension | AAM | T_N_ | T_CM_ | T_EM_ | T_EMRA_ | T_N_ | T_CM_ | T_EM_ | T_EMRA_ | T_N_ | T_CM_ | T_EM_ | T_EMRA_ | T_N_ | T_CM_ | T_EM_ | T_EMRA_ | T_N_ | T_CM_ | T_EM_ | T_EMRA_ | T_N_ | T_CM_ | T_EM_ | T_EMRA_ |
|  |  |  |  | 66.7  11.9  66.3  70  64.4  78.6  65.3  78.9  36.8  65.223.631.127.127.722.982.5 | 22.2  14.3  18.3  19.1  24.8  13.3  24.9  15.8  42.1  14.223  21.638.928.722  7.02 | 0  73.8  7  3.18  4.84  4.34  4.89  5.26  21.1  18.647.344.631.836.651.46.43 | 11.1  0  8.33  7  5.98  3.76  4.89  0  0  2.03  6.06  2.7  2.18  6.93  3.67  4.09 | 33.3  36.4  57.4  66.7  57.1  44.1  48.4  10  22.2  7.6914.844.68.788.398.4557 | 0  9  12.8  13  25.2  15.3  34.1  0  33.3  26.518.317.822.748.326.814 | 33.3  54.5  9.93  7.25  8.4  18.6  9.89  0  44.4  65  62.729.763.440.664.822.4 | 33.3  0  19.9  13  9.24  22  7.69  0  0  0.85  4.23  7.92  5.14  2.8  0  6.54 | 33.3  36.4  44.9  76.9  53.6  49.4  53.2  88.2  17.4  14.718.812.58.967.6916.461.3 | 0  9  21.3  12.8  23.7  11.8  17  5.88  56.5  37.923.98.3325.742.341  17.3 | 66.7  54.5  21.3  3.85  11.3  21.2  14.9  5.88  26.1  47.456.879.255.648.737.717.3 | 0  0  12.6  6.41  11.3  17.6  14.9  0  0  0  0.57  0  9.7  1.28  4.92  4 | 33.3  0  82.4  12.5  12.5  14.5  25.9  68.4  37.5  45.241.423.134.133.317.147.1 | 0  0  0  0  0  0  0  0  0  4.766.9  7.69  5.736.060  0 | 0  0  0  0  25  7.25  11.1  10.5  0  21.40  38.53.2342.428.617.6 | 66.7  0  17.6  87.5  62.5  78.3  63  21.1  62.5  28.6  51.7  30.8  57  18.2  54.3  35.3 | 0  100  33.3  50  0  15  18.2  82.4  0  80  53.340  33.643.80  60.9 | 0  0  0  0  0  0  0  0  0  0  0  0  0.93  0  6.258.7 | 0  0  0  0  0  10  36.4  0  0  8  20  60  10.3  43.825  4.35 | 0  0  66.7  50  100  75  45.5  17.6  0  12  26.7  0  55.1  12.5  68.8  17.4 | 0  0  70  71.4  0  10.9  50  81.8  40  40  67.757.139.248  21.161.5 | 0  0  20  0  0  0  0  0  0  0  0  0  3.8  24  0  15.4 | 0  0  0  0  12.5  15.2  12.5  9.09  0  20  0  28.6  2.5316  10.50 | 100  0  10  28.6  87.5  73.9  37.5  9.09  60  40  32.3  14.3  54.4  12  68.4  23.1 |
| M  F  F  F  M  M  F  M  F  M  F  M | 45  31  59  57  38  36  55  44  55  34  36  49 | Hypertension  Hypertension | AMM | 73.9  0  13.6  65.3  69  13.5  17.610.851.626.750  78.9 | 26.1  50  27.3  15.5  15.6  26.1  47.727  26.646.5284.93 | 0  50  54.5  10  6.62  56.5  25.940.518.822.11814.8 | 0  0  4.55  8.92  8.75  3.86  8.81  21.6  3.12  6.98  4  1.41 | 44.4  0  20  46.2  38.4  6.94  20.933.341.529.134.828 | 0  25  40  25.8  22.3  18.1  37.233.329.332.732.614 | 44.4  75  30  14.4  18.8  74.3  37.233.324.427.330.450 | 11.1  0  10  13.6  20  0  4.65  0  4.88  10.9  4.35  8 | 0  0  48.6  35.5  3.28  X  9.380  50  35.634.134.5 | 33.3  12.5  23.4  30  10  X  33.914.333.339.734.120.7 | 33.3  87.5  13.1  17.2  84.4  X  51.685.70  19.224.431 | 33.3  0  14.9  17.2  1.64  X  5.21  0  16.7  5.48  7.32  13.8 | 100  66.7  4.26  20  46.7  13.3  57.946.621.943.339.570.2 | 0  0  0  4  0  0  2.380  1.041.926.980 | 0  0  38.3  12  0  5.33  9.522.7418.811.522.10 | 0  33.3  57.4  64  53.3  81.3  30.2  50.7  58.3  43.3  31.4  29.8 | 0  0  1.64  31.6  50  12.3  60.649  32.5  48.550  60.9 | 0  0  0  0  25  0  0.520  3.97  1.942.270 | 0  0  36.1  5.26  12.5  3.51  9.335.7312.713.615.94.35 | 100  0  62.3  63.2  12.5  84.2  29.5  45.2  50.8  35.9  31.8  34.8 | 50  3.85  40.9  50  0  X  55.265  28.145.858.882.9 | 0  0  0  0  0  X  1.230  0  2.08  0  0 | 0  34.6  22.7  13.6  4.88  X  16  3.756.7418.829.40 | 50  61.5  36.4  36.4  95.1  X  27.6  31.2  65.2  33.3  11.8  17.1 |
| M  F  F  M  M  F  M  F  F  M | 41  43  54  44  34  57  35  37  47  51 | Hyperlipidemia | MMM | 41.3  71.6  26.864.448.235.729.67.2552.439.6 | 33.6  21.5 30.624.432.217  35.621.745.916.4 | 13.8  3.29  39.37.7816.238.330  65.21.7340.3 | 11.3  3.68  3.28  3.33  3.25  8.94  4.72  5.8  0  3.77 | 31.1  47.1  37.536.727.328.735.48.7  24  15.1 | 25.5  23  30.88.1630.224.432.327.548  23.7 | 24.5  13.8  25  22.4  36.639.431.563.828  59.1 | 18.9  16.1  8.65  32.7  5.85  7.5  0.77  0  0  2.15 | 28.5  71.1  17.912.526.227.826.411.523.942.9 | 32.6  11.9  30.812.534.226.633.338.544.225.2 | 18.6  3.54  51.350  32.738  35.644.928.328.6 | 20  13.5  0  25  6.93  7.59  4.6  5.13  3.54  3.36 | 4.52  13.6  25  34.17.0453.444.650  60  12.3 | 3.87  2.27  2.5  2.257.040.680  0  0  3.08 | 27.1  9.09  15  1.13  14.11.358.930  0  33.8 | 64.5  75  57.5  62.5  71.8  44.6  46.4  50  40  50.8 | 11.2  15.8  39.136.127.257.557.650  50  22.2 | 2.24  0  0  2.231.941.252.020  25  0 | 29.1  26.3  9.784.4616.54.3814.10  0  66.7 | 57.5  57.9  51.1  57.2  54.4  36.9  26.3  50  25  11.1 | 13.7  45.2  36.730.415.559  47.640  28.552.9 | 3.43  3.23  0  1.795.63  0  0  20  3.695.88 | 20.6  6.45  20  3.57  15.50  7.94  0  9.135.88 | 62.3  45.2  43.3  64.3  63.4  41  44.4  40  58.7  35.3 |
